# A heralded and error-rejecting three-photon hyper-parallel quantum gate through cavity-assisted interactions

**DOI:** 10.1038/s41598-018-20148-z

**Published:** 2018-01-30

**Authors:** Ji-Zhen Liu, Hai-Rui Wei, Ning-Yang Chen

**Affiliations:** School of Mathematics and Physics, University of Science and Technology Beijing, Beijing, 100083 China

## Abstract

Hyper-parallel quantum computation is a promising and fruitful area of research with its high capacity and low loss rate characters. In this paper, we propose a heralded, compact, scalable, and deterministic error-rejecting scheme for implementing three-photon hyper-parallel Toffoli gate simultaneously acting on polarization and spatial degrees of freedom. It is a practical and unity gate without strong coupling strength limitations, since the undesired performances caused by the side leakage and the limited coupling strength are detected by the single-photon detectors. The success of our proposal can be heralded by the detectors, and the efficiency can be further improved by repeating the operation processes when the detectors are clicked. The evaluation of gate performance with experimental parameters shows that it is feasible with current experimental technology.

## Introduction

Exploiting quantum mechanics and the superposition principle, quantum computers outperform classical computers on certain computationally demanding problems like searching databases^1,2^, factoring large integers^3^, quantum simulation and modeling^4^. Nowadays, much research has been done in traditional parallel quantum computing^5,6^. Recently, hyper-parallel quantum information processing (QIP) has attracted growing interest^7,8^ duo to its high capacity encoding, low loss rate, increased security of the communication, and hyper-parallel computing. Quantum conditional gates^9,10^ or similar logic operations^11^ are the key components for quantum computing^12^. Moreover, multi-qubit conditional gates are very useful in quantum error correction^13^, quantum algorithm^14^, fault-tolerant quantum circuits^15^, and quantum network^16^. Controlled-NOT (CNOT) gate is the most popular universal conditional gate^12^, i.e., supplemented with one-qubit unitary gates one can perform any quantum computing^9^. Implementation of multi-qubit Toffoli gate^17^ or Fredkin gate^18^ is an important milestone for scalable quantum computer. Therefore, investigation of the hyper-parallel multi-qubit gate will open an avenue in scalable hyper-parallel quantum information processing.

Impressive theoretical and experimental progress has been made and realized in parallel and hyper-parallel CNOT, Toffoli, and Fredkin gates in various physical systems today^19–24^. Photon has been recognized as one of the most popular and promising candidates for parallel and especially hyper-parallel quantum information processing thanks to its available single-qubit operations, low decoherence, faithful transmission of information, and many available qubit-like degrees of freedom (DOFs)^25^. However, scalability is the main objective for optical computing with current technology due to weak interaction in single-photon level. One approach for achieving this goal is to employ linear optics and photon detectors, and only probabilistic gates with a maximal probability of success 3/4 have been explored^19^. Another approach is to employ distributed or modular architecture. The emitted solid-state platform can be efficiently configured to mediate photon-photon or spin-spin interaction. Nowadays, the efficient emitted platforms have ranged from natural atoms^26^ or Rydbery atom ensembles^27^ to artificial atoms such as QD^28,29^, diamond nitrogen vacancy defect center^30^, and superconductor^21^. The interactions between individual photons and stationary qubits are generally weak, and cavity quantum electrodynamics (QED) are usually exploited to overcome this challenge by confining photons for a long time in a small region^31^.

Numerous theoretical and experimental achievements about the emitter-based gates have been reported^32,33^. Recently, quantum dot (QD) inside a microcavity has received much attention because of its *μ*s coherence time^34,35^, fast QD spin manipulation^36^, scalability, and optical property. In 2008, Hu *et al*.^28,29^ proposed an interesting QD-based emitter, a self-assembled In(Ga)AS QD or GaAs interface QD confined in a resonant microcavity, and such emitter was experimentally demonstrated in 2011 and 2016, respectively^37,38^. Based on QD-emitter, Bonato *et al*.^39^, Deng *et al*.^40^, and Wang *et al*.^41^ proposed schemes for implementing hybrid, electronic, and photonic quantum computing gate. Other applications such as hyper-parallel universal gates^42,43^, repeater^44^, photonic transistor^45^, router^46^, entanglement and hyper-entanglement states analysis^47^, purification and distillation^48^ and so on have been proposed. The side leakage and the imperfect birefringent propagation of the incident photon, which reduce the fidelity and efficiency of the devices, are not taken into account in above schemes.

Hyperentanglement is a potential resource in QIP, and it can be used for some important applications^49^. In 2016, Li and Deng^50^ proposed a scheme for generating error-rejecting Bell states assisted by QD-single-side-cavity platforms. Hyper-parallel CNOT and Toffoli gates with perfect input-output relations have been proposed^42,51^. In this paper, we propose a deterministic scheme for efficiently implementing self-error-rejecting optical hyper-parallel Toffoli gate on polarization and spatial DOFs, without using any auxiliary polarization DOFs. The imperfect birefringent propagations of the incident photons induced by side leakage and the limited QD-cavity coupling strength are taken into account. Our practical scheme has some characters. First, it is a practical proposal, and the inevitable imperfect performances can be detected by single-photon detectors. Second, the strong coupling limitations can be avoided and the proposal allows low-*Q* cavities. Third, the near-unity fidelity can be achieved in principle. Fourth, the success of the deterministic scheme can be heralded by the single-photon detectors. Fifth, the efficiency of the scheme can be further improved by repeating the operation processes when the detectors are clicked. Sixth, compared to the traditional one, our proposal reduces the noise effect, operation time, and quantum resources by a half.

## Results

### The optical property of a QD-microcavity platform

**Figure 1:**
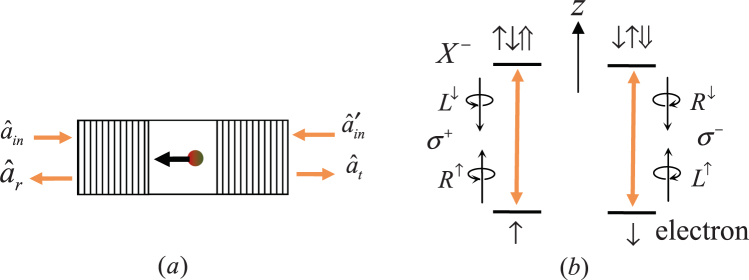
(**a**) A schematic diagram of the quantum dot-cavity coupled system. (**b**) Schematic description of the spin-dependent optical transition rules. |*L*^↑^〉 (|*L*^↓^〉) and |*R*^↑^〉 (|*R*^↓^〉) represent the left- and right-handed circularly polarized photons parallelled (antiparallelled) with the growth axis, respectively. |↑〉 and |↓〉 represent the electron spin states with $|\pm \frac{1}{2}\rangle$, respectively. $|\Uparrow \rangle$ and $|\Downarrow \rangle$ represent the heavy-hole spin states with $|\pm \frac{3}{2}\rangle$, respectively.

Now we consider a singly charged QD [e.g., a self-assembled in(Ga)As QD or GaAs QD] incorporated into the center of a double-sided optical resonator with tens to hundreds GHz (high speed)^29,52^. As shown in Fig. 1, the QD is confined in the center of the double-sided microcavity. When an excess electron is injected, a negative charged exciton (*X*^−^) consisting of two electrons and a hole is created by optical excitation^53^. Here, both the ground states (the electron spin states) and the excited states (the spin states of the *X*^−^) are twofold degenerate due to the Kramer’s theorem^54,55^. There are two spin-dependent transition interacting with circularly polarized lights in QD-doubled-sided unite^56^ due to the Pauli’s exclusion principle and the conservation of the total spin angular momentum. The *s*_*z*_ = +1 polarized photon (marked by |*L*^↓^〉 and |*R*^↑^〉) and *s*_*z*_ = −1 polarized photon (marked by |*L*^↑^〉 and |*R*^↓^〉) only couple the transition $|\uparrow \rangle \to |\uparrow \downarrow \Uparrow \rangle$ and $|\downarrow \rangle \to |\downarrow \uparrow \Downarrow \rangle$, respectively, and then the incident photons are reflected by the cavity. Otherwise, the photons can not couple to the QD and feel the cold cavity (g = 0), and then it is transmitted through the cavity. Upon reflection, both the polarization and the propagation direction of the incident photon will be flipped. Whereas, the spin of the electron remains unchanged upon both the reflection and transmission. Here, |*R*〉 and |*L*〉 denote the right- and left-handed circularly polarized light, respectively. The superscripts |↑〉 and |↓〉 of |*M*^↑^〉 and |*M*^↓^〉 indicate the propagation direction of the *M*-polarized photon parallel and antiparallel to the *z* axis (the growth and optical axis). $|\Uparrow \rangle$ and $|\Downarrow \rangle$ represent the heavy-hole spin states with $|\pm \frac{3}{2}\rangle$, respectively. |↑〉 and |↓〉 represent the electron spin states with $|\pm \frac{1}{2}\rangle$, respectively.

The reflection and the transmission coefficients of double-sided cavity can be obtained from the Heisenberg equations of motion for the cavity field operator $\hat{a}$, the (*X*^−^) dipole operator *σ*_−_ and the input-output relations between the output fields ${\hat{a}}_{r}$, ${\hat{a}}_{t}$, and the input fields ${\hat{a}}_{in}$, ${\hat{a}^{\prime} }_{in}$^57^,

1 $$\begin{array}{rcl}\frac{d\hat{a}}{dt} & = & -[i({\omega }_{c}-\omega )+\kappa +\frac{{\kappa }_{s}}{2}]\,\hat{a}-{\rm{g}}{\sigma }_{-}-\sqrt{\kappa }\,{\hat{a}}_{in}-\sqrt{\kappa }\,{\hat{a}^{\prime} }_{in}+\hat{H},\\ \frac{d{\sigma }_{-}}{dt} & = & -[i({\omega }_{{X}^{-}}-\omega )+\frac{\gamma }{2}]\,{\sigma }_{-}-{\rm{g}}{\sigma }_{z}\hat{a}+\hat{G},\\ {\hat{a}}_{r} & = & {\hat{a}}_{in}+\sqrt{\kappa }\,\hat{a},\\ {\hat{a}}_{t} & = & {\hat{a}^{\prime} }_{in}+\sqrt{\kappa }\,\hat{a}.\end{array}$$

where *ω*, *ω*_*c*_, and ${\omega }_{{X}^{-}}$ denote the frequencies of the external field (probe photon), the cavity mode, and the *X*^−^ transition, respectively. g denotes the cavity coupling strength between *X*^−^ and cavity mode. *γ*/2, *κ*, and *κ*_*s*_/2 denote the decay rates of the *X*^−^ dipole, the cavity field, and the leaky mode (side leakage), respectively. $\hat{H}$ and $\hat{G}$ are the noise operators.

In the approximation of a weak excitation condition with (*X*^−^) predominantly staying in the ground state, and taking 〈*σ*_*z*_〉 ≈ −1 and ${\sigma }_{z}\hat{a}=-\hat{a}$, one can find that the reflection and the transmission coefficients of the (*X*^−^) cavity system can be expressed as^29,58^,

2 $$r(\omega )=1+t(\omega ),\quad t(\omega )=\frac{-\kappa \,[i({\omega }_{{X}^{-}}-\omega )+\frac{\gamma }{2}]}{[i({\omega }_{{X}^{-}}-\omega )+\frac{\gamma }{2}]\,[i({\omega }_{c}-\omega )+\kappa +\frac{{\kappa }_{s}}{2}]+{{\rm{g}}}^{2}}.$$

For convenience to discussions, we consider the cavity mode is resonant with optical transition of the QD, i.e., ${\omega }_{c}={\omega }_{{X}^{-}}={\omega }_{0}$, and then the interaction between the incident photon and the cavity can be summarized as

3 $$\begin{array}{ll}|{R}^{\uparrow }\uparrow \rangle \to r|{L}^{\downarrow }\uparrow \rangle , & |{L}^{\downarrow }\uparrow \rangle \to r|{R}^{\uparrow }\uparrow \rangle ,\\ |{R}^{\uparrow }\downarrow \rangle \to {t}_{0}|{R}^{\uparrow }\downarrow \rangle , & |{L}^{\downarrow }\downarrow \rangle \to {t}_{0}|{L}^{\downarrow }\downarrow \rangle ,\\ |{R}^{\downarrow }\downarrow \rangle \to r|{L}^{\uparrow }\downarrow \rangle , & |{L}^{\uparrow }\downarrow \rangle \to r|{R}^{\downarrow }\downarrow \rangle ,\\ |{R}^{\downarrow }\uparrow \rangle \to {t}_{0}|{R}^{\downarrow }\uparrow \rangle , & |{L}^{\uparrow }\uparrow \rangle \to {t}_{0}|{L}^{\uparrow }\uparrow \rangle .\end{array}$$

However, in the practical working, the imperfect birefringence of the cavity, caused by the nonzero photon bandwidth, the mismatch and the finite coupling rate between the photon and the cavity mode, makes Eq. (3) changed as

4 $$\begin{array}{ll}|{R}^{\uparrow }\uparrow \rangle \to r|{L}^{\downarrow }\uparrow \rangle +t|{R}^{\uparrow }\uparrow \rangle , & |{L}^{\downarrow }\uparrow \rangle \to r|{R}^{\uparrow }\uparrow \rangle +t|{L}^{\downarrow }\uparrow \rangle ,\\ |{R}^{\uparrow }\downarrow \rangle \to {t}_{0}|{R}^{\uparrow }\downarrow \rangle +{r}_{0}|{L}^{\downarrow }\downarrow \rangle , & |{L}^{\downarrow }\downarrow \rangle \to {t}_{0}|{L}^{\downarrow }\downarrow \rangle +{r}_{0}|{R}^{\uparrow }\downarrow \rangle ,\\ |{R}^{\downarrow }\downarrow \rangle \to r|{L}^{\uparrow }\downarrow \rangle +t|{R}^{\downarrow }\downarrow \rangle , & |{L}^{\uparrow }\downarrow \rangle \to r|{R}^{\downarrow }\downarrow \rangle +t|{L}^{\uparrow }\downarrow \rangle ,\\ |{R}^{\downarrow }\uparrow \rangle \to {t}_{0}|{R}^{\downarrow }\uparrow \rangle +{r}_{0}|{L}^{\uparrow }\uparrow \rangle , & |{L}^{\uparrow }\uparrow \rangle \to {t}_{0}|{L}^{\uparrow }\uparrow \rangle +{r}_{0}|{R}^{\downarrow }\uparrow \rangle .\end{array}$$

Here the reflection coefficient *r* (*r*_0_) and the transmission coefficient *t* (*t*_0_) are described by Eq. (2) with g ≠ 0 (g = 0).

In the following, we introduce our practical proposal for implementing emission-based three-photon hyper-parallel controlled-controlled-phase-flip gate, step by step. It is known that Toffoli gate is equivalent to the controlled-controlled-phase-flip gate upon to two Hadamard gates acting on the target qubit.

### Three-photon Toffoli gate acting on polarization DOF

**Figure 2:**
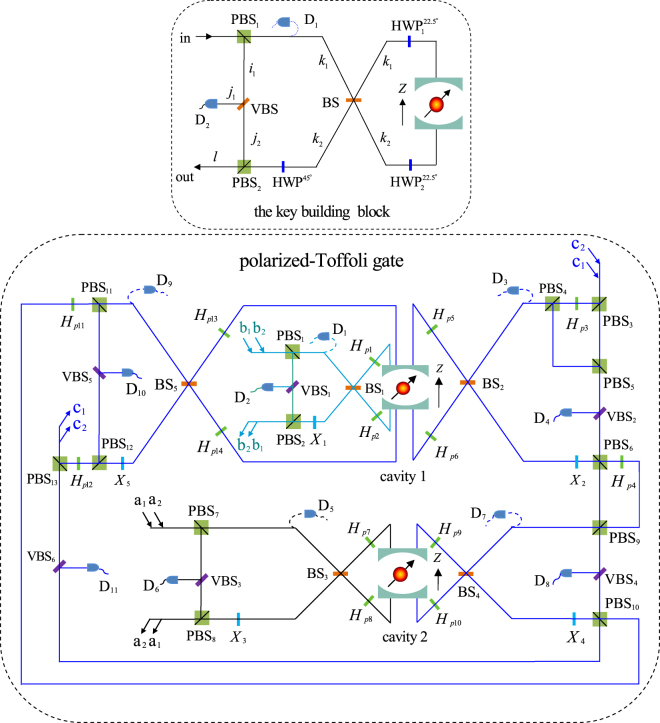
A schematic diagram for implementing a polarized-Toffoli gate assisted by double-sided microcavities. PBS_*j*_ (*j* = 1, …, 13), a circularly polarizing beam splitter, which is used to transmit the *R*-polarized wave packets and reflect the *L*-polarized wave packets. BS_*j*_ (*j* = 1, … 5), balanced nonpolarizing beam splitter, transforms the input modes as ${k}_{1}\to ({k}_{1}+{k}_{2})/\sqrt{2}$, ${k}_{2}\to ({k}_{1}-{k}_{2})/\sqrt{2}$. VBS_*j*_ (*j* = 1, …, 6), an adjustable beam splitter with transmission coefficient *t* − *t*_0_ and reflection coefficient $\sqrt{1-{(t-{t}_{0})}^{2}}$. D_*i*_ (*i* = 1, 2, …, 11), a single photon detector. *H*_*pj*_ (*j* = 1, 2, …, 14) represents a Hadamard operation on polarization, and it can be completed by using a half wave plate rotating at 22.5°. *X*_*j*_ (*j* = 1, 2, …, 5) represents a bit-flip operation on polarization, and it can completed by using a half wave plate rotating at 45°.

Let us first introduce the performance of the key building block in our scheme. As shown in Fig. 2(a), the circularly polarizing beam splitter, PBS_1_, transmits the input *R*-polarized wave packet into spatial mode *k*_1_ and reflects the *L*-polarized wave packet into spatial mode *i*_1_. Before and after the wave packets emitted from spatial mode *k*_1_ and *k*_2_ interact with the QD, Hadamard operations are performed on the spatial mode and the polarized mode respectively. The performance of the polarized- (spatial-) Hadamard operation can be written as

5 $$H=\frac{1}{\sqrt{2}}\,(\begin{array}{ll}1 & 1\\ 1 & -1\end{array}).$$

in the basis {|*R*〉, |*L*〉} ({|*k*_1_〉, |*k*_2_〉}). Here, the spatial- and polarized- Hadamard transformation, *H*_*s*_ and *H*_*p*_, can be implemented by using a nonpolarizing beam splitter (BS) and a half wave plate oriented at 22.5°, respectively. Therefore, operations (PBS_1_ → BS → *H*_*p*1_, *H*_*p*2_ → QD → *H*_*p*1_, *H*_*p*2_ → BS) make the joint state

6 $$|{\varphi }_{1}\rangle =(\alpha |R\rangle +\beta |L\rangle )\,(\gamma |\uparrow \rangle +\delta |\downarrow \rangle )$$

become

7 $$\begin{array}{rcl}|{\varphi }_{2}\rangle  & = & \alpha \gamma (r+{t}_{0})|{R}^{{k}_{1}}\rangle |\uparrow \rangle +\alpha \gamma (t-{t}_{0})|{L}^{{k}_{2}}\rangle |\uparrow \rangle +\alpha \delta (r+{t}_{0})|{R}^{{k}_{1}}\rangle |\downarrow \rangle \\  &  & -\alpha \delta (t-{t}_{0})|{L}^{{k}_{2}}\rangle |\downarrow \rangle +\beta \gamma |{L}^{{i}_{1}}\rangle |\uparrow \rangle +\beta \delta |{L}^{{i}_{1}}\rangle |\downarrow \rangle .\end{array}$$

Subsequently, the bit-flip operation *X*, which can be implemented by using a half wave plate oriented at 45°, is performed on the wave packet emitted from the spatial mode *k*_2_. The wave packet emitted from the spatial mode *i*_1_ passes through the adjustable beam splitter, VBS, with transmission coefficient *t* − *t*_0_ and reflection coefficient $\sqrt{1-{(t-{t}_{0})}^{2}}$. And then, the wave packets emitted from the spatial mode *k*_2_ and *j*_2_ arrive at PBS_2_, simultaneously. Therefore, *X*, VBS, and PBS_2_ make Eq. (7) changed as

8 $$\begin{array}{rcl}|{\varphi }_{3}\rangle  & = & \alpha \gamma (t-{t}_{0})|{R}^{{k}_{2}}\rangle |\uparrow \rangle -\alpha \delta (t-{t}_{0})|{R}^{{k}_{2}}\rangle |\downarrow \rangle \\  &  & +\beta \gamma (t-{t}_{0})|{L}^{{j}_{2}}\rangle |\uparrow \rangle +\beta \delta (t-{t}_{0})|{L}^{{j}_{2}}\rangle |\downarrow \rangle \\  &  & +\alpha \gamma (r+{t}_{0})|{R}^{{k}_{1}}\rangle |\uparrow \rangle +\alpha \delta (r+{t}_{0})|{R}^{{k}_{1}}\rangle |\downarrow \rangle \\  &  & +\beta \gamma \sqrt{1-{(t-{t}_{0})}^{2}}|{L}^{{j}_{1}}\rangle |\uparrow \rangle \\  &  & +\beta \delta \sqrt{1-{(t-{t}_{0})}^{2}}|{L}^{{j}_{1}}\rangle |\downarrow \rangle .\end{array}$$

If detectors *D*_1_ and *D*_2_ are not clicked, the state of the system in Eq. (8) is collapsed into the desired state

9 $$\begin{array}{rcl}|{\varphi }_{4}\rangle  & = & \alpha \gamma (t-{t}_{0})|{R}^{l}\rangle |\uparrow \rangle -\alpha \delta (t-{t}_{0})|{R}^{l}\rangle |\downarrow \rangle \\  &  & +\beta \gamma (t-{t}_{0})|{L}^{l}\rangle |\uparrow \rangle +\beta \delta (t-{t}_{0})|{L}^{l}\rangle |\downarrow \rangle .\end{array}$$

From Eqs (5–9), when the detectors are not clicked, the transformation of the building block composed of PBS_1_, PBS_2_, BS, VBS, *H*_*p*1_, *H*_*p*2_, QD, *X*, *D*_1_, and *D*_2_, can be written as

10 $${U}_{block}=(t-{t}_{0})\,(\begin{array}{llll}1 & 0 & 0 & 0\\ 0 & -1 & 0 & 0\\ 0 & 0 & 1 & 0\\ 0 & 0 & 0 & 1\end{array}),$$

in the basis {|*R* ↑〉, |*R* ↓〉, |*L* ↑〉, |*L* ↓〉}. The efficiency of the key building block can be further improved by repeating the operation processes when the detectors are clicked.

Now we introduce the performance of our polarized-Toffoli gate, step by step. As shown in Fig. 2(b), suppose that the initial states of gate photons *a*, *b*, and *c* are prepared as |*φ*_*a*_〉, |*φ*_*b*_〉, |*φ*_*c*_〉, respectively. Electron mediums 1 and 2 are initially prepared in the states |↓_1_〉 and |↓_2_〉, respectively. Here,

11 $$|{\phi }_{a}\rangle =|{\phi }_{a}{\rangle }_{p}\otimes |{\phi }_{a}{\rangle }_{s},\quad |{\phi }_{b}\rangle =|{\phi }_{b}{\rangle }_{p}\otimes |{\phi }_{b}{\rangle }_{s},\quad |{\phi }_{c}\rangle =|{\phi }_{c}{\rangle }_{p}\otimes |{\phi }_{c}{\rangle }_{s},$$

with

12 $$\begin{array}{cc}|{\phi }_{a}{\rangle }_{p}={\alpha }_{1}|{R}_{1}\rangle +{\alpha }_{2}|{L}_{1}\rangle , & |{\phi }_{b}{\rangle }_{p}={\beta }_{1}|{R}_{2}\rangle +{\beta }_{2}|{L}_{2}\rangle ,\\ |{\phi }_{c}{\rangle }_{p}={\gamma }_{1}|{R}_{3}\rangle +{\gamma }_{2}|{L}_{3}\rangle , & |{\phi }_{a}{\rangle }_{s}={\epsilon }_{1}|{a}_{1}\rangle +{\epsilon }_{2}|{a}_{2}\rangle ,\\ |{\phi }_{b}{\rangle }_{s}={\varepsilon }_{1}|{b}_{1}\rangle +{\varepsilon }_{2}|{b}_{2}\rangle , & |{\phi }_{c}{\rangle }_{s}={\zeta }_{1}|{c}_{1}\rangle +{\zeta }_{2}|{c}_{2}\rangle .\end{array}$$

Where *a*_1_ and *a*_2_ (*b*_1_, *b*_2_ or *c*_1_, *c*_2_) represent the two spatial modes of the photon *a* (*b* or *c*). *α*_1_, *α*_2_, *β*_1_, *β*_2_, *γ*_1_, *γ*_2_, $\epsilon$_1_, $\epsilon$_2_, *ε*_1_, *ε*_2_, *ζ*_1_, and *ζ*_2_ are complex coefficients satisfying |*α*_1_|^2^ + |*α*_2_|^2^ = 1, |*β*_1_|^2^ + |*β*_2_|^2^ = 1, |*γ*_1_|^2^ + |*γ*_2_|^2^ = 1, $|{\varepsilon }_{1}{|}^{2}+|{\varepsilon }_{2}{|}^{2}=1$, $|{\epsilon }_{1}{|}^{2}+|{\epsilon }_{2}{|}^{2}=1$, and |*ζ*_1_|^2^ + |*ζ*_2_|^2^ = 1.

First, photon *b* in the spatial modes *b*_1_ and *b*_2_ is injected. Before and after photon *b* passes through the building block composed of PBS_1_, PBS_2_, VBS_1_, BS_1_, *H*_*p*1_, *H*_*p*2_, QD_1_, *X*_1_, *D*_1_, and *D*_2_, Hadamard operations, *H*_*e*1_ and *H*_*e*2_, are performed on the QD_1_ with a single photon, or an ultrafast ps or fs ${(\tfrac{\pi }{2})}_{y}$ optical pulse from the cavity side^34,36,59^. When detectors *D*_1_ and *D*_2_ are not clicked, the above operations (*H*_*e*1_ → building block → *H*_*e*2_) transform the system composed of photon *a*, *b*, *c*, and QD_1_ and QD_2_ from the initial state |*φ*_0_〉 into |*φ*_1_〉. Here,

13 $$|{\phi }_{0}\rangle =|{\phi }_{a}{\rangle }_{p}\otimes |{\phi }_{a}{\rangle }_{s}\otimes |{\phi }_{b}{\rangle }_{p}\otimes |{\phi }_{b}{\rangle }_{s}\otimes |{\phi }_{c}{\rangle }_{p}\otimes |{\phi }_{c}{\rangle }_{s}\otimes |{\downarrow }_{1}\rangle \otimes |{\downarrow }_{2}\rangle ,$$

14 $$\begin{array}{rcl}|{\phi }_{1}\rangle  & = & (t-{t}_{0})\,({\alpha }_{1}|{R}_{1}\rangle +{\alpha }_{2}|{L}_{1}\rangle )\,({\beta }_{1}|{R}_{2}{\uparrow }_{1}\rangle +{\beta }_{2}|{L}_{2}{\downarrow }_{1}\rangle )\,({\gamma }_{1}|{R}_{3}\rangle \\  &  & +{\gamma }_{2}|{L}_{3}\rangle )|{\downarrow }_{2}\rangle \otimes |{\phi }_{a}{\rangle }_{s}\otimes |{\phi }_{b}{\rangle }_{s}\otimes |{\phi }_{c}{\rangle }_{s}.\end{array}$$

Second, photon *c* in spatial modes *c*_1_ and *c*_2_ is injected and arrives at PBS_3_. PBS_3_ transmits the *R*_3_-polarized wave packet to PBS_5_ and reflects the *L*_3_-polarized wave packet into the building block composed of PBS_4_, VBS_2_, BS_2_, *H*_*p*5_, *H*_*p*6_, QD_1_, *X*_2_, PBS_6_, *D*_3_, and *D*_4_. Before and after the wave packets interact with the block, Hadamard operations, *H*_*p*3_ and *H*_*p*4_, are performed on it, respectively. When *D*_1,…,4_ are not clicked, operations (PBS_3_ → PBS_5_ → *H*_*p*3_ → building block → *H*_*p*4_) make |*φ*_1_〉 become

15 $$\begin{array}{rcl}|{\phi }_{2}\rangle  & = & {(t-{t}_{0})}^{2}\,({\alpha }_{1}|{R}_{1}\rangle +{\alpha }_{2}|{L}_{1}\rangle )\,({\beta }_{1}{\gamma }_{1}|{R}_{2}{R}_{3}{\uparrow }_{1}\rangle +{\beta }_{1}{\gamma }_{2}|{R}_{2}{L}_{3}{\uparrow }_{1}\rangle \\  &  & +{\beta }_{2}{\gamma }_{1}|{L}_{2}{R}_{3}{\downarrow }_{1}\rangle -{\beta }_{2}{\gamma }_{2}|{L}_{2}{R}_{3}{\downarrow }_{1}\rangle )|{\downarrow }_{2}\rangle \otimes |{\phi }_{a}{\rangle }_{s}\otimes |{\phi }_{b}{\rangle }_{s}\otimes |{\phi }_{c}{\rangle }_{s}.\end{array}$$

Third, photon *a* in the spatial modes *a*_1_ or *a*_2_ is launched into the building block composed of PBS_7_, VBS_3_, BS_3_, *H*_*p*7_, *H*_*p*8_, QD_2_, *X*_3_, PBS_8_, *D*_5_, and *D*_6_. Before and after the wave packet interacts with the block, *H*_*e*3_ and *H*_*e*4_ are performed on QD_2_, respectively. When *D*_1,…,6_ are not clicked, operations (*H*_*e*3_ → building block → *H*_*e*4_) make |*φ*_2_〉 changed as

16 $$\begin{array}{rcl}|{\phi }_{3}\rangle  & = & {(t-{t}_{0})}^{3}\,({\alpha }_{1}|{R}_{1}{\uparrow }_{2}\rangle +{\alpha }_{2}|{L}_{1}{\downarrow }_{2}\rangle )\,({\beta }_{1}{\gamma }_{1}|{R}_{2}{R}_{3}{\uparrow }_{1}\rangle +{\beta }_{1}{\gamma }_{2}|{R}_{2}{L}_{3}{\uparrow }_{1}\rangle \\  &  & +{\beta }_{2}{\gamma }_{1}|{L}_{2}{R}_{3}{\downarrow }_{1}\rangle -{\beta }_{2}{\gamma }_{2}|{L}_{2}{R}_{3}{\downarrow }_{1}\rangle )\otimes |{\phi }_{a}{\rangle }_{s}\otimes |{\phi }_{b}{\rangle }_{s}\otimes |{\phi }_{c}{\rangle }_{s}.\end{array}$$

Fourth, photon *c* is leaded into the building block composed of PBS_9_, VBS_4_, BS_4_, *H*_*p*9_, *H*_*p*10_, QD_2_, *X*_4_, PBS_10_, *D*_7_, and *D*_8_. If *D*_1,…,8_ are not clicked, the joint state is collapsed into

17 $$\begin{array}{rcl}|{\phi }_{4}\rangle  & = & {(t-{t}_{0})}^{4}\,[{\alpha }_{1}|{R}_{1}{\uparrow }_{2}\rangle \,({\beta }_{1}{\gamma }_{1}|{R}_{2}{R}_{3}{\uparrow }_{1}\rangle +{\beta }_{1}{\gamma }_{2}|{R}_{2}{L}_{3}{\uparrow }_{1}\rangle \\  &  & +{\beta }_{2}{\gamma }_{1}|{L}_{2}{R}_{3}{\downarrow }_{1}\rangle -{\beta }_{2}{\gamma }_{2}|{L}_{2}{R}_{3}{\downarrow }_{1}\rangle )+{\alpha }_{2}|{L}_{1}{\downarrow }_{2}\rangle \,({\beta }_{1}{\gamma }_{1}|{R}_{2}{R}_{3}{\uparrow }_{1}\rangle \\  &  & +{\beta }_{1}{\gamma }_{2}|{R}_{2}{L}_{3}{\uparrow }_{1}\rangle +{\beta }_{2}{\gamma }_{1}|{L}_{2}{R}_{3}{\downarrow }_{1}\rangle \\  &  & +{\beta }_{2}{\gamma }_{2}|{L}_{2}{R}_{3}{\downarrow }_{1}\rangle )]\otimes |{\phi }_{a}{\rangle }_{s}\otimes |{\phi }_{b}{\rangle }_{s}\otimes |{\phi }_{c}{\rangle }_{s}.\end{array}$$

Fifth, after photon *c* passes through VBS_6_ and the building block composed of PBS_11_, VBS_5_, BS_5_, *H*_*p*13_, *H*_*p*14_, QD_1_, *X*_5_, PBS_12_, *D*_9_, and *D*_10_, the desired wave packets are mixed at PBS_13_. If *D*_1,…,11_ are not clicked, the state of the system is then becoming

18 $$\begin{array}{rcl}|{\phi }_{5}\rangle  & = & {(t-{t}_{0})}^{5}\,[{\alpha }_{1}|{R}_{1}{\uparrow }_{2}\rangle \,({\beta }_{1}{\gamma }_{1}|{R}_{2}{R}_{3}{\uparrow }_{1}\rangle \\  &  & +{\beta }_{1}{\gamma }_{2}|{R}_{2}{L}_{3}{\uparrow }_{1}\rangle +{\beta }_{2}{\gamma }_{1}|{L}_{2}{R}_{3}{\downarrow }_{1}\rangle \\  &  & +{\beta }_{2}{\gamma }_{2}|{L}_{2}{L}_{3}{\downarrow }_{1}\rangle )+{\alpha }_{2}|{L}_{1}{\downarrow }_{2}\rangle \,({\beta }_{1}{\gamma }_{1}|{R}_{2}{R}_{3}{\uparrow }_{1}\rangle \\  &  & +{\beta }_{1}{\gamma }_{2}|{R}_{2}{L}_{3}{\uparrow }_{1}\rangle +{\beta }_{2}{\gamma }_{1}|{L}_{2}{R}_{3}{\downarrow }_{1}\rangle \\  &  & -{\beta }_{2}{\gamma }_{2}|{L}_{2}{L}_{3}{\downarrow }_{1}\rangle )]\otimes |{\phi }_{a}{\rangle }_{s}\otimes |{\phi }_{b}{\rangle }_{s}\otimes |{\phi }_{c}{\rangle }_{s}.\end{array}$$

Sixth, the QD_1_ and QD_2_ are measured in the basis $\{|\pm \rangle =(|\uparrow \rangle \pm |\downarrow \rangle )/\sqrt{2}\}$. On detecting the QD_1_ and QD_2_ in the state |+_1_〉 and |+_2_〉, one disentangled |*φ*_5_〉 into the desired outcomes of the polarized-Toffoli gate, that is,

19 $$\begin{array}{rcl}|{\phi }_{6}\rangle  & = & {(t-{t}_{0})}^{5}\,[{\alpha }_{1}|{R}_{1}\rangle \,({\beta }_{1}{\gamma }_{1}|{R}_{2}{R}_{3}\rangle +{\beta }_{1}{\gamma }_{2}|{R}_{2}{L}_{3}\rangle \\  &  & +{\beta }_{2}{\gamma }_{1}|{L}_{2}{R}_{3}\rangle +{\beta }_{2}{\gamma }_{2}|{L}_{2}{L}_{3}\rangle )\\  &  & +{\alpha }_{2}|{L}_{1}\rangle \,({\beta }_{1}{\gamma }_{1}|{R}_{2}{R}_{3}\rangle +{\beta }_{1}{\gamma }_{2}|{R}_{2}{L}_{3}\rangle \\  &  & +{\beta }_{2}{\gamma }_{1}|{L}_{2}{R}_{3}\rangle -{\beta }_{2}{\gamma }_{2}|{L}_{2}{L}_{3}\rangle )]\otimes |{\phi }_{a}{\rangle }_{s}\otimes |{\phi }_{b}{\rangle }_{s}\otimes |{\phi }_{c}{\rangle }_{s}.\end{array}$$

As for the |+_1_〉 and |−_2_〉 case, classical feed-forward single-qubit operations *σ*_*z*_ = |*R*〉 〈*R*| − |*L*〉 〈*L*|, which can be implemented by using half wave plate oriented at 0°, are performed on the outing photon *a* to lead the outcomes to the desired state described by Eq. (19). As for the |−_1_〉 and |+_2_〉 case, *σ*_*z*_s are performed on the outing photon *b*. As for the |−_1_〉 and |−_2_〉 case, *σ*_*z*_s are performed on the outing photon *b* and *a*, respectively.

Putting all the pieces together, one can see that Fig. 2(b) can deterministically implement an error-rejecting three-photon polarized-Toffoli gate in a heralded way, without any influence on their spatial mode quantum states.

### Three-photon Toffoli gate acting on spatial DOF

Up to now, we have discussed the implementation of the error-rejecting polarized-Toffoli gate without any influence on their spatial mode quantum states. In order to implement a hyper-parallel Toffoli gate performing controlled-controlled-NOT operations on the polarization and spatial DOFs, independently, a scheme for implementing error-rejecting three-photon spatial-Toffoli gate without any negative influence on their polarization states will be designed in this subsection.

**Figure 3:**
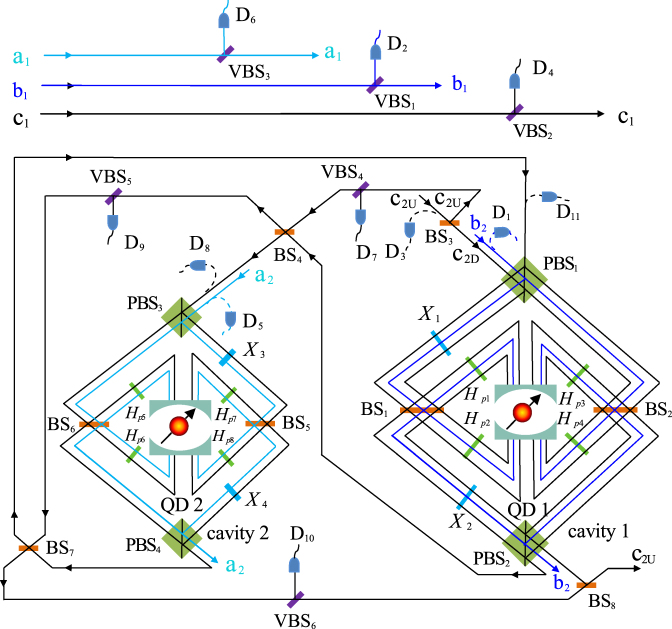
A schematic diagram for a spatial-Toffoli gate assisted by double-sided microcavities. Adjustable beam splitter, VBS_1_ and VBS_3,_…_,6_, with transmission coefficient *t* − *t*_0_ and reflection coefficient $\sqrt{1-{(t-{t}_{0})}^{2}}$. VBS_2_ with transmission coefficient (*t* − *t*_0_)^3^ and reflection coefficient $\sqrt{1-{(t-{t}_{0})}^{6}}$.

The scheme we designed for implementing the spatial-Toffoli gate without influence on their polarization states is depicted by Fig. 3, and it can be completed by six steps. The three photons *a*, *b*, and *c* are initially prepared in the arbitrary product states with polarization and spatial DOFs (see Eq. (11)). QD_1_ and QD_2_ are prepared in the states |↑_1_〉 and |↓_2_〉, respectively.

First, the photon *b* is injected. The wave packets emitted from the spatial mode *b*_1_ arrive at VBS_1_ with transmission coefficient *t* − *t*_0_ and reflection coefficient $\sqrt{1-{(t-{t}_{0})}^{2}}$. While the ones emitted from the spatial mode *b*_2_ arrive at PBS_1_, and PBS_1_ transmits the *R*-polarized wave packet to the right round composed of BS_2_, *H*_*p*3_, *H*_*p*4_, QD_1_ and reflects the *L*-polarized wave packet to the left round composed of *X*_1_, BS_1_, *H*_*p*1_, *H*_*p*2_, QD_1_, *X*_2_. Here the two rounds complete the transformations

20 $$\begin{array}{ll}|R\uparrow \rangle \mathop{\to }\limits^{{\rm{rounds}}}(t-{t}_{0})|L\uparrow \rangle , & |R\downarrow \rangle \mathop{\to }\limits^{{\rm{rounds}}}-(t-{t}_{0})|L\downarrow \rangle ,\\ |L\uparrow \rangle \mathop{\to }\limits^{{\rm{rounds}}}(t-{t}_{0})|R\uparrow \rangle , & |L\downarrow \rangle \mathop{\to }\limits^{{\rm{rounds}}}-(t-{t}_{0})|R\downarrow \rangle .\end{array}$$

It is noted that before and after the wave packets interact with QD_1_, *H*_*e*_s are performed on QD_1_, respectively. Therefore, when *D*_1_ and *D*_2_ are not clicked, operations (VBS_1_, PBS_1_ → BS_2_ → *H*_*p*3_, *H*_*e*_ → QD_1_ → *H*_*p*4_, *H*_*e*_ → PBS_2_ and VBS_1_, PBS_1_ → *X*_1_ → BS_1_ → *H*_*p*1_, *H*_*e*_ → QD_1_ → *H*_*p*2_, *H*_*e*_ → *X*_2_ → PBS_2_) transform the system composed of photons *a*, *b*, *c* and QD_1_, QD_2_ from |*ψ*_0_〉 = |*φ*_*a*_〉 ⊗ |*φ*_*b*_〉 ⊗ |*φ*_*c*_〉 ⊗ |↑_1_〉 ⊗ |↓_2_〉 into

21 $$\begin{array}{rcl}|{\psi }_{1}\rangle  & = & (t-{t}_{0})\,({\epsilon }_{1}|{a}_{1}\rangle +{\epsilon }_{2}|{a}_{2}\rangle )\,({\varepsilon }_{1}|{b}_{1}{\uparrow }_{1}\rangle +{\varepsilon }_{2}|{b}_{2}{\downarrow }_{1}\rangle )\,({\zeta }_{1}|{c}_{1}\rangle \\  &  & +{\zeta }_{2}|{c}_{2}\rangle )|{\downarrow }_{2}\rangle \otimes |{\phi }_{a}{\rangle }_{p}\otimes |{\phi }_{b}{\rangle }_{p}\otimes |{\phi }_{c}{\rangle }_{p}.\end{array}$$

Second, photon *c* emitted from the spatial mode *c*_1_ passes through VBS_2_ with transmit (*t* − *t*_0_)^3^ and reflect $\sqrt{1-{(t-{t}_{0})}^{6}}$. Photon *c* emitted from the spatial mode *c*_2*D*_ passes through the round composed of PBS_1_, *X*_1_, *H*_*p*1_, *H*_*p*2_, BS_1_, *X*_2_, QD_1_, PBS_2_ or the round composed of PBS_1_, *H*_*p*3_, *H*_*p*4_, BS_2_, QD_1_, PBS_2_. Before and after the photon passes through the two rounds, Hadamard operation *H*_*s*_s, which complete the transformation,

22 $$\begin{array}{l}|{c}_{2U}\rangle \to \frac{1}{\sqrt{2}}(|{c}_{2U}\rangle +|{c}_{2D}\rangle ),\quad |{c}_{2D}\rangle \to \frac{1}{\sqrt{2}}(|{c}_{2U}\rangle -|{c}_{2D}\rangle \mathrm{)}.\end{array}$$

are performed on the spatial mode of photon *c* by BS_1_ and BS_2_. It is worthy to note that after BS_3_, only the wave packets emitted from the spatial mode *c*_2*D*_ interact with QD_1_. When *D*_3_ and *D*_4_ are not clicked, |*ψ*_1_〉 is changed as

23 $$\begin{array}{rcl}|{\psi }_{2}\rangle  & = & ({\epsilon }_{1}|{a}_{1}\rangle +{\epsilon }_{2}|{a}_{2}\rangle )\otimes |{\downarrow }_{2}\rangle \otimes |{\phi }_{a}{\rangle }_{p}\\  &  & \otimes |{\phi }_{b}{\rangle }_{p}\otimes |{\phi }_{c}{\rangle }_{p}\otimes [(t-{t}_{0}{)}^{4}{\varepsilon }_{1}{\zeta }_{1}|{b}_{1}{c}_{1}\rangle |{\uparrow }_{1}\rangle \\  &  & +{(t-{t}_{0})}^{2}{\varepsilon }_{1}{\zeta }_{2}|{b}_{1}{c}_{2U}\rangle |{\uparrow }_{1}\rangle +{(t-{t}_{0})}^{4}{\varepsilon }_{2}{\zeta }_{1}|{b}_{2}{c}_{1}\rangle |{\downarrow }_{1}\rangle \\  &  & +{(t-{t}_{0})}^{2}{\varepsilon }_{2}{\zeta }_{2}|{b}_{2}{c}_{2D}\rangle |{\downarrow }_{1}\rangle \mathrm{]}.\end{array}$$

Third, photon *a* emitted from the spatial mode *a*_1_ passes through VBS_3_ with transmit (*t* − *t*_0_), and the one emitted from the spatial mode *a*_2_ passes through the round composed of PBS_3_, *X*_3_, *H*_*p*7_, *H*_*p*8_, BS_5_, *X*_4_, QD_2_, PBS_4_ or the round composed of PBS_3_, *H*_*p*5_, *H*_*p*6_, BS_6_, QD_2_, PBS_4_. Here, before and after the photon passes through the two rounds, *H*_*e*_s are performed on QD_2_, respectively. When *D*_5_ and *D*_6_ are not clicked, |*ψ*_2_〉 is collapsed into the desired state

24 $$\begin{array}{rcl}|{\psi }_{3}\rangle  & = & ({\epsilon }_{1}|{a}_{1}\rangle |{\downarrow }_{2}\rangle +{\epsilon }_{2}|{a}_{2}\rangle |{\uparrow }_{2}\rangle )\otimes |{\phi }_{a}{\rangle }_{p}\otimes |{\phi }_{b}{\rangle }_{p}\\  &  & \otimes |{\phi }_{c}{\rangle }_{p}\otimes [(t-{t}_{0}{)}^{5}{\varepsilon }_{1}{\zeta }_{1}|{b}_{1}{c}_{1}\rangle |{\uparrow }_{1}\rangle \\  &  & +{(t-{t}_{0})}^{3}{\varepsilon }_{1}{\zeta }_{2}|{b}_{1}{c}_{2U}\rangle |{\uparrow }_{1}\rangle +{(t-{t}_{0})}^{5}{\varepsilon }_{2}{\zeta }_{1}|{b}_{2}{c}_{1}\rangle |{\downarrow }_{1}\rangle \\  &  & +{(t-{t}_{0})}^{3}{\varepsilon }_{2}{\zeta }_{2}|{b}_{2}{c}_{2D}\rangle |{\downarrow }_{1}\rangle \mathrm{]}.\end{array}$$

Fourth, photon *c* emitted from the spatial mode *c*_2*D*_ interacts with the round composed of PBS_3_, *X*_3_, *H*_*p*7_, *H*_*p*8_, BS_5_, *X*_4_, QD_2_, PBS_4_ or the round composed of PBS_3_, *H*_*p*5_, *H*_*p*6_, BS_6_, QD_2_, PBS_4_. From Eq. (20), one can see that when *D*_7_, *D*_8_, and *D*_9_ are not clicked, the two rounds induce |*ψ*_3_〉 to be

25 $$\begin{array}{rcl}|{\psi }_{4}\rangle  & = & {(t-{t}_{0})}^{4}\,\{{\epsilon }_{1}|{a}_{1}\rangle |{\downarrow }_{2}\rangle \,[(t-{t}_{0}){\varepsilon }_{1}{\zeta }_{1}|{b}_{1}{c}_{1}\rangle |{\uparrow }_{1}\rangle +{\varepsilon }_{1}{\zeta }_{2}|{b}_{1}{c}_{2U}\rangle |{\uparrow }_{1}\rangle \\  &  & +(t-{t}_{0}){\varepsilon }_{2}{\zeta }_{1}|{b}_{2}{c}_{1}\rangle |{\downarrow }_{1}\rangle -{\varepsilon }_{2}{\zeta }_{2}|{b}_{2}{c}_{2D}\rangle |{\downarrow }_{1}\rangle ]\\  &  & +{\epsilon }_{2}|{a}_{2}\rangle |{\uparrow }_{2}\rangle \,[(t-{t}_{0}){\varepsilon }_{1}{\zeta }_{1}|{b}_{1}{c}_{1}\rangle |{\uparrow }_{1}\rangle \\  &  & +{\varepsilon }_{1}{\zeta }_{2}|{b}_{1}{c}_{2U}\rangle |{\uparrow }_{1}\rangle +(t-{t}_{0}){\varepsilon }_{2}{\zeta }_{1}|{b}_{2}{c}_{1}\rangle |{\downarrow }_{1}\rangle \\  &  & +{\varepsilon }_{2}{\zeta }_{2}|{b}_{2}{c}_{2D}\rangle |{\downarrow }_{1}\rangle ]\}\\  &  & \otimes |{\phi }_{a}{\rangle }_{p}\otimes |{\phi }_{b}{\rangle }_{p}\otimes |{\phi }_{c}{\rangle }_{p}.\end{array}$$

Fifth, after wave packets emitted from the spatial *c*_2*U*_ and *c*_2*D*_ are mixed at BS_7_, the wave packet emitted from *c*_2*U*_ passes through the block composed of PBS_1_, PBS_2_, *X*_1_, *X*_2_, BS_1_, BS_2_, *H*_*p*1_, *H*_*p*2_, *H*_*p*3_, and *H*_*p*4_, and then they are mixed with the wave packet emitted from *c*_2*D*_ passing through the VBS_6_ and BS_8_. When *D*_9_, *D*_10_ and *D*_11_ are not clicked, one can see that |*ψ*_4_〉 is collapsed into the desired state

26 $$\begin{array}{rcl}|{\psi }_{5}\rangle  & = & {(t-{t}_{0})}^{5}\,\{{\epsilon }_{1}|{a}_{1}\rangle |{\downarrow }_{2}\rangle [{\varepsilon }_{1}{\zeta }_{1}|{b}_{1}{c}_{1}\rangle |{\uparrow }_{1}\rangle \\  &  & +{\varepsilon }_{1}{\zeta }_{2}|{b}_{1}{c}_{2U}\rangle |{\uparrow }_{1}\rangle +{\varepsilon }_{2}{\zeta }_{1}|{b}_{2}{c}_{1}\rangle |{\downarrow }_{1}\rangle \\  &  & +{\varepsilon }_{2}{\zeta }_{2}|{b}_{2}{c}_{2U}\rangle |{\downarrow }_{1}\rangle ]\\  &  & +{\epsilon }_{2}|{a}_{2}\rangle |{\uparrow }_{2}\rangle \,[{\varepsilon }_{1}{\zeta }_{1}|{b}_{1}{c}_{1}\rangle |{\uparrow }_{1}\rangle +{\varepsilon }_{1}{\zeta }_{2}|{b}_{1}{c}_{2U}\rangle |{\uparrow }_{1}\rangle \\  &  & +{\varepsilon }_{2}{\zeta }_{1}|{b}_{2}{c}_{1}\rangle |{\downarrow }_{1}\rangle -{\varepsilon }_{2}{\zeta }_{2}|{b}_{2}{c}_{2U}\rangle |{\downarrow }_{1}\rangle ]\}\otimes |{\phi }_{a}{\rangle }_{p}\otimes |{\phi }_{b}{\rangle }_{p}\otimes |{\phi }_{c}{\rangle }_{p}.\end{array}$$

Sixth, we measure the QD_1_ and QD_2_ in the basis $\{|\pm \rangle =(|\uparrow \rangle \pm |\downarrow \rangle )/\sqrt{2}\}$. If the outcomes of the QD_1_ and QD_2_ are |+_1_〉 and |+_2_〉, respectively, and then the desired performance is completed. As for the |+_1_〉 and |−_2_〉 case, phase shifter *e*^*iπ*^, which completes the transformation |*R*〉 → −|*R*〉 and |*L*〉 → −|*L*〉, is performed on the spatial mode *a*_1_ to complete the spatial-Toffoli gate. As for the |−_1_〉 and |+_2_〉 case, phase shifter *e*^*iπ*^ is performed on the spatial mode *b*_2_. As for the |−_1_〉 and |−_2_〉 case, phase shifter *e*^*iπ*^s are performed on the spatial mode *a*_1_ and *b*_2_, respectively. That is to say, measurement and the classical feed-forward single-qubit operations make |*ψ*_5_〉 become

27 $$\begin{array}{rcl}|{\psi }_{6}\rangle  & = & {(t-{t}_{0})}^{5}\,\{{\epsilon }_{1}|{a}_{1}\rangle \,[{\varepsilon }_{1}{\zeta }_{1}|{b}_{1}{c}_{1}\rangle +{\varepsilon }_{1}{\zeta }_{2}|{b}_{1}{c}_{2U}\rangle \\  &  & +{\varepsilon }_{2}{\zeta }_{1}|{b}_{2}{c}_{1}\rangle +{\varepsilon }_{2}{\zeta }_{2}|{b}_{2}{c}_{2U}\rangle ]\\  &  & +{\epsilon }_{2}|{a}_{2}\rangle [{\varepsilon }_{1}{\zeta }_{1}|{b}_{1}{c}_{1}\rangle +{\varepsilon }_{1}{\zeta }_{2}|{b}_{1}{c}_{2U}\rangle \\  &  & +{\varepsilon }_{2}{\zeta }_{1}|{b}_{2}{c}_{1}\rangle -{\varepsilon }_{2}{\zeta }_{2}|{b}_{2}{c}_{2U}\rangle ]\}\\  &  & \otimes |{\phi }_{a}{\rangle }_{p}\otimes |{\phi }_{b}{\rangle }_{p}\otimes |{\phi }_{c}{\rangle }_{p}.\end{array}$$

From Eqs (20–27), one can see that the quantum circuit shown in Fig. 3 can be used to implement a robust three-photon spatial-Toffoli gate without any influence on their polarization mode quantum states in a deterministic way.

## Discussion

QDs mimic the behavior of single atomic dipole-like transitions. However, unlike atoms, QDs can be easily incorporated into solid-state devices such as cavities or waveguide that enhance the light-matter interaction for scalable QIP. Moreover, the manipulation can be achieved with high-speed (up to THz). The coupling efficiency of the pillar microcavity is higher than waveguide as the pillar cavity mode is Guassian type and matches perfectly with the external laser beam. Compared to the devices via QD-double-sided emitters, the ones via QD-single-sided emitters are fragile due to the balanced reflectance, for the coupled and uncoupled cavities are necessary to get high fidelity^29^. The length and deep of the gates via QD-double-sided emitters usually are surpass the ones via QD-single-sided emitters as the maximum Faraday rotation *π* can be achieved in particular QD-double-sided emitter^29^, whereas *π*/2 is for the QD-single-sided oemitter^60^.

The unconstructed low theoretical lower bound for a generic traditional *n*-qubit gates is $[\tfrac{1}{4}{\mathrm{(4}}^{n}-3n-\mathrm{1)}]$ CNOT gates^61^. Up to now, the optimal cost of a traditional Toffoli gate acting on single DOF is 6 CNOT gates^12,62^. The scheme we designed for physical implementing a hyper-parallel Toffoli gate acting on two DOFs, and the essential cost of our scheme is 6 CNOT gates. Compared to the traditional one, our hyper-parallel Toffoli gate reduces the quantum resource, the operation time, and the influence of the noise by half. The previous works mainly were investigated via the optical transition rules in Eq. (3), that is, the side-leakage and the imperfect birefringence of the cavity are not taken into account^28,29,37^ and *t*_0_ → −1, *r* → 1. Our hyper-parallel Toffoli gate are constructed via Eq. (4), and the undesired performances caused by the side leakage and the imperfect birefringence are detected by the detectors. Moreover, the strong coupling limitation can be avoided in our scheme because the fidelity of our scheme is unity in principle.

In summary, we have designed a quantum circuit for implementing a heralded error-rejecting hyper-parallel Toffoli gate assisted by QD-double-sided cavities. It is a practical proposal, and the side leakage and the imperfect birefringence of the incident photons are detected. The fidelity of the present gate is always unity and the efficiency can be further improved by repeating the construction processes. In addition, our scheme overcomes the exciting strong coupling limitations, and can work in both the strong-coupling and the weak-coupling regimes. These interesting features make us believe that the present work may be useful for some practical hyper-parallel quantum information processing, including hyper-entanglement concentration^63^ and purification^48^, hyper-entanglement state analysis, hyper-parallel quantum repeater and so on.

### Evaluation of the performance

An efficient QD-photon has been recognized as a potential building block for QIP due to their inherent scalability and mature semiconductor technology^28,29^. QDs incorporated into photonic-crystal (PC) waveguides^64,65^, photonic nanowires^66^, pillar microcavities^37,67,68^, and photonic crystal cavities cite^69^ have been achieved in experiment. In the present, the double-sided symmetric pillar cavity supports circularly polarized light, and it incorporates a negative charged QD. This type of cavity has been fabricated in experiment^68^. Some specific symmetry photonic crystal nanocavities^70^ are suitable for proposal as well. Compared to weak coupling cavity-QED system, strong coupling cavity-QED system is a challenge in experiment with current technology. Fortunately, the strong coupling regime *g*/(*κ* + *κ*_*s*_) = 2.4 (*g* = 80 *μ*eV, *κ* + *κ*_*s*_ = 33 *μ*eV), has been demonstrated for INAs QDs in the state-of-the-art pillar microcavity recently^67,71^. The strong limitations are not necessary in our proposal because the imperfect performance caused by the strong coupling can be detected by the detector. Hu *et al*.^29,37^ showed that the *π* phase shift, which is necessary for the emitter-based quantum gates, can be achieved in this particular double-sided-QD-cavity combination.

**Figure 4:**
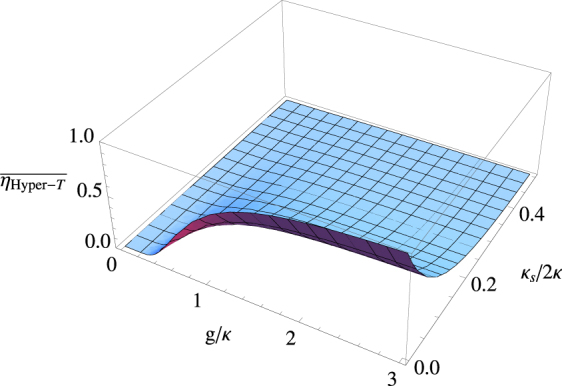
The average efficiency of the hyper-parallel Toffoli gate as the function of the coupling strength *g*/*κ* and side leakage *κ*_*s*_/*κ* averaged over [0, 2*π*]. *γ* = 0.1*κ* is taken.

The performance of the gate can be characterized by the fidelity and efficiency. According to the arguments in result, one can find that the unity fidelity of our practical hyper-parallel Toffoli gate can be achieved in principle if the overall phase is ignored. The efficiency of the present gate depends on cavity QED parameters (*g*, *κ*, *κ*_*s*_, *γ*), and it can be further improved by repeating the operation processes when the undesired performances are detected. Here, the efficiency is defined as the ratio between the number of the output photons to the input photons. The shape functions $\overline{\eta }$ is plotted in Fig. 4 for the average efficiency of the present gate averaged over [0, 2*π*]. *γ* ~ *μ*eV caused by the the spontaneous emission and the pure dephasing is usually smaller than *κ* + *κ*_*s*_ in high-quality QD-cavity samples. Here *γ* = 0.1*κ*, which is experimentally achieved, is taken. Figure 4 suggests that we could make high efficiency in the strong coupling regime when the side leakage *κ*_*s*_/*κ* is small. The strong coupling QD-cavity system has been reported^67,71^. The side leakage, *κ*_*s*_, can be reduced by engineering the fabrication and various cavity details such as materials, structures, size, etc.

Photon loss has no contribution to the fidelity of the gate. In realistic case, the fidelity of the emitter can be decreased by a factor^29^, $F=[1+\exp \,(-{\rm{\Delta }}t/{T}_{2}^{e}\mathrm{)]/2}$, due to spin decoherence. Therefore, the lower is the ${\rm{\Delta }}t/{T}_{2}^{e}$, the higher is the *F*. Here ${T}_{2}^{e}$ and Δ*t* are the electron spin coherence time and the time interval between input photons, respectively. ${T}_{2}^{e}\sim \mu s$ and Δ*t* ~ ns have been achieved in experiment^72^. The imperfect spin-selection rules, induced by the heavy-light hole mixing, reduce emitter fidelity by a few percent^73,74^. The heavy-light hole mixing depends on the kind, shape and size of the QD. The emitter fidelity can be reduced by amount of *F* = [1 − exp(−*τ*/*T*_2_)] caused by the QD spin dephasing, and such influence can be neglected because *T*_2_ ~ *μ*s and *τ* ~ ps have been experimentally demonstrated in the coupling regime with 10^4^–10^5^ ^75,76^. Here, *τ* and *T*_2_ are the cavity photon lifetime and QD coherence time, respectively. Moreover, the technical imperfection such as photon detection events, the unbalanced PBSs, and the spatial mismatch between the cavity mode and the photon influence the success probability and the fidelity of the gate.
